# Three-dimensional diamond planar spiral detectors

**DOI:** 10.1038/s41598-025-93332-7

**Published:** 2025-03-12

**Authors:** Rebecca J. Watkins, Patrick S. Salter, Ralph J. Moors, Richard B. Jackman

**Affiliations:** 1 London Centre for Nanotechnology and Department of Electronic and Electrical Engineering, UCL (University College London), 17-19 Gordon Street, London, WC1H 0AH UK; 2 Department of Engineering Science, University of Oxford, Parks Road, Oxford, OX1 3PJ UK

**Keywords:** Electrical and electronic engineering, Nanoscale devices, Nanosensors, Nuclear fusion and fission, Sensors and biosensors

## Abstract

Diamond’s superior carrier transport properties and unparalleled radiation tolerance make it an ideal material for alpha/neutron detection. High performing diamond detectors are already commercially available. However, even high quality single crystal diamond can degrade after high doses of radiation, resulting in a reduction in carrier mean free path. It is well known that reducing the carrier collection distance, by decreasing the detector electrode spacing, makes radiation detectors more tolerant of mean free path reduction, and therefore more resilient to high radiation doses. One approach for thin device fabrication involves using thin diamond substrates, which can be fragile. In this work, a thin detector has been fabricated using a thick, highly resilient 300 μm diamond substrate by utilising a 3D network of laser-written nano-carbon network electrodes. An optimised femto-second laser write process, utilising specialised optical arrangements, is used to realise planar configured diamond detectors, comprising two Ti/Pt/Au spiral electrodes, connected to internal spiral nano-carbon network ’wall’ electrodes, which extend 20 μm below the surface and have a 50 μm separation. It was found that introducing the nano-carbon network electrodes greatly improved the detector resolution and Charge Collection Efficiency. With close to 100% charge collection efficiency and ns rise times demonstrated, achieving “thin” detector performance, in “thick”, structural substrates.

## Introduction

Alpha particle detectors are crucial for the operation and decommissioning of nuclear power plants. In these environments they are exposed to large doses of high energy alphas, neutrons and other forms of radiation^1^. Robust, high resolution, high sensitivity and low background detectors are an absolute necessity.

Diamond solid-state detectors are excellent alternatives to conventional scintillation and gas filled detectors. Diamond’s wide band-gap and large carrier mobility results in reduced leakage currents, fast signal collection^2^ and its radiation tolerance is unmatched by other semiconductors^3^. Furthermore, its low atomic number leads to a low capture cross section for gamma radiation, which reduces background noise caused by gamma-decay of activated materials or secondary decay of alpha particles. Diamond detectors have been used for alpha spectroscopy as far back as the 1970s^4^. Early diamond detectors utilised polycrystalline (PC) CVD diamond, leading to low charge collection efficiencies (CCE) due to charge trapping at grain boundaries and non uniform electric fields. The most success was achieved with carefully selected natural diamond, permitting CCEs of 100%^5^.

In recent years, techniques for growing commercially viable free-standing single-crystal (SC) diamond emerged and ‘electronic grade’ (ppb N) diamond in sizes up to 30 mm in diameter, are available. This has enabled the production of commercially available diamond radiation detectors (CIVIDEC). With numerous works demonstrating full charge collection, high resolution and fast response times (as low as 260 ps reported in^6^) for both alpha^2,7,8^ and neutron detection^9–14^.

The most common detector type is the MIM (Metal Insulator Metal) detector, which consists of an intrinsic diamond substrate sandwiched between two electrodes^15^. Alpha particles enter the detector through the top electrode and interact with the diamond, generating electron hole pairs (EHPs). A DC bias is applied across the detector and the EHPs move in the direction of the applied electric field, producing current. In order to achieve full charge collection, these detectors usually require large operating bias’ and careful selection of high quality SC diamond substrates. Additionally, alpha particles have a short penetration depth in all materials. For this reason MIM detectors are not desirable, as there is considerable energy absorption even in a thin metal top contact, which does not result in EHP generation, and therefore reduces CCE and resolution. Thus, planar detectors tend to be preferable. These consist of both electrodes on the alpha-facing surface, with EHP’s being separated transverse to the depth of the detector region and the radiation direction.

Furthermore, future high energy physics experiments and fusion reactors demand greater radiation fluences and ${>10^{16}}$ particles $\mathrm {cm^{-2}}$^16,17^ are expected. The RD42 collaboration conducted a study to measure the radiation tolerance of CVD diamond against protons, ions and neutrons of various energies^16,18,19^, and showed that at fluences of $\mathrm {10^{17}\,cm^{-2}}$ the mean drift path of electron hole pairs reduced to 16 μm and the detector becomes trap limited, reducing the effective charge collection distance.

Thus innovative design approaches are needed to reduce the detector electrode spacing, L. One approach involves etching the diamond substrate to achieve an extremely thin membrane. Detectors as thin as μm have been achieved^7^, with close to 100% CCE. However thin membranes present challenges with regards to packaging the detector for use in harsh environments.

Here, an alternative approach is presented, involving fabricating a planar detector with internal electrodes, achieved using a pulsed laser, where the detector thickness is defined by the distance between internal electrodes rather than the substrate thickness. Diamond detectors for charged particle detection^20,21^, tracking^22–24^ and x-ray imaging^25^, have already been demonstrated using this approach. However, initial works were limited by the laser fabrication process, since it is difficult to fabricate the internal electrodes in close proximity to each other and the surface. This led to the need for large L and also limits the available geometries.

More recently an improved fabrication technique has been developed by the authors, which utilises a fs laser and adaptive optic elements to correct for aberrations in the diamond lattice. This method has enabled the production of conductive tracks with low resistivity and complex geometries^26^. These tracks have been termed Nano-carbon Networks (NCNs) due to the presence of various carbon forms, confirmed using high-resolution transmission electron microscopy^27^. Using this technology, detectors with close to 100% CCE have been demonstrated for particle tracking and X-ray imaging^22,23,25^ purposes. Furthermore, their durability in $\mathrm {3.5 \times 10^{15} \, p \, cm^{-2}}$, has also been demonstrated^18^. However less attention has been paid to their use for alpha detection purposes.

In this work planar configured detectors were fabricated, comprising two Ti/Pt/Au spiral electrodes separated by 35 μm on the alpha-facing surface of the diamond substrate. Similar structures, often referred to as planar detectors, have been adopted in numerous works^22,23,28,29^ but tend to demonstrate lower CCE when compared to MIM, membrane or three-dimensional detector architectures due to non uniform field distributions. Therefore three-dimensional internal NCN wall electrodes, following the same spiral pattern, chosen to optimise the uniformity of the electric field, were also fabricated underneath the Ti/Pt/Au surface contacts.

The fabrication of the NCN electrodes was as follows. First, a two-dimensional spiral NCN wire was fabricated, using a pulsed fs laser. The write process starts 20 μm below the surface, and builds up layer by layer to produce a wall height of 20 μm, reaching the top surface. This depth was chosen to correspond with the penetration depth of the test alpha particles. It is possible to increase the wall depth when designing a detector for other types of more penetrating radiation. Futhermore, it is envisioned that in the future the internal NCN electrodes could be contacted via the back of the substrate using NCN through vias, permitting removal of metal contacts from the alpha-facing surface, a known point of failure for detectors exposed to large doses of gamma radiation^30^.

The fabricated detectors were characterised. Firstly with Current-Voltage measurements and Raman spectroscopy. Secondly with alpha spectroscopy and transient current measurements during exposure to alpha particles from a sealed ^241^Am (Americium-241) source.

They have also been compared to a reference MIM detector, the CIVIDEC B3 Spectroscopic detector (500 μm thick, operating at 400 V). To confirm the quality of this detector, transient current measurements were performed during exposure to ^241^Am alpha particles and the corresponding data is shown in S4. The mobility and saturation velocities were extracted and found to be typical of high quality diamond substrates operated at high field^8,31^.

## Results

### Alpha spectroscopy measurements

**Fig. 1:**
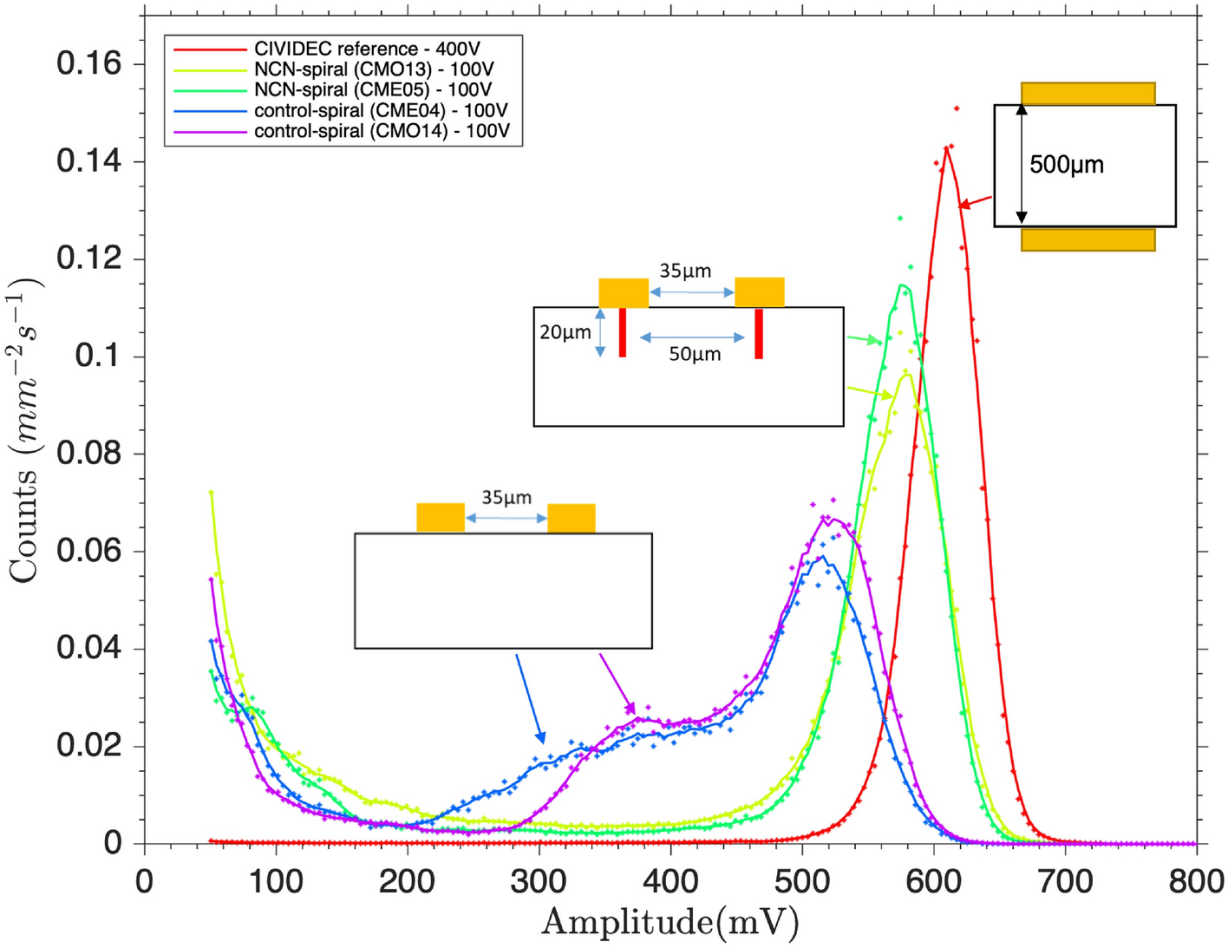
A comparison of the ^241^Am alpha spectrum of diamond spiral detectors with and without internal nano-carbon network spiral electrodes; and a CIVIDEC reference detector.

The fabricated spiral detectors were first primed with ^241^Am alpha particles until they reached maximum efficiency. This has been discussed in more detail in the methods section and the resulting change in the ^241^Am alpha spectrum with time, is presented in S1 and S2. The summed alpha spectra for all the fabricated spiral detectors were compared with the CIVIDEC MIM detector, and can be seen in Fig. 1.

It is clear that incorporating the NCN “wall” electrodes has improved the detector performance, characterised by improved resolution and greater CCE (calculated by comparing the position of the peak compared with the CIVIDEC detector). With the NCN-spiral detectors demonstrating a 9% greater CCE than the control-spiral detectors, shown in Table 1. The error values provided in Table 1 were determined using the full width half maximum (FWHM) of the alpha spectrum peak, where error = FWHM/(2ln(2)).

It can also be seen that the CIVIDEC MIM detector appears to have a larger CCE than the fabricated NCN-spiral detector. However, it is expected that the shaping output of the CIVIDEC Cx-L spectroscopic amplifier was not well matched to the NCN-spiral detector, leading to loss of some of the signal. The authors considered converting amplitude to energy to account for this loss, however this would require many assumptions to be made to define the EHP generation energy. Thus, it was preferred to keep the x-axis as amplitude, so that the reader can appreciate that the chosen amplifier can greatly impact the observed CCE. TCT measurements, presented below, use a more device agnostic amplification circuit, and so can be used for direct device comparison.

**Table 1:** Table displaying the ^241^Am alpha peak amplitude and counts for all detectors, and the calculated detector efficiency.

Detectors	Peak amplitude (mV)	Normalised count rate ($\mathrm {mm^{-2}s^{-1}}$)	Efficiency ($\%$)
CIVIDEC MIM	610 ± 20	0.148 ± 0.001	100 ± 5
NCN-spiral (CMO13)	580 ± 30	0.103 ± 0.001	94 ± 6
NCN-spiral (CME05)	580 ± 30	0.121 ± 0.001	94 ± 6
control-spiral (CME04)	520 ± 40	0.061 ± 0.001	85 ± 7
control-spiral (CMO14)	520 ± 40	0.069 ± 0.001	85 ± 7

### Transient current measurements

Transient Current measurements were also performed for all fabricated spiral detectors after alpha priming. For these measurements, a different amplifier was used (C2-TCT Broadband Amplifier) and the raw (un-shaped) transient current signals were measured for operating bias’s between 40 and 120 V. A representative signal for the NCN-spiral detectors and the CIVIDEC detector can be seen in S3 a) and b) respectively. The signal shape for the fabricated detectors is different to the CIVIDEC Detector. They display a thin transient current signal, rather than a wide rectangular shaped signal, which is expected since the electrode spacing is much smaller. They also display oscillations in the signal, which are characteristic of poor impedance matching.

For MIM detectors with a large electrode spacing, it is assumed that EHPs are generated in the vicinity of the top electrode, thus the velocity of electrons and holes can be extracted using the positive and negative transient current signals, as shown in S4. The velocities can then be plotted against field, allowing extraction of the electron and hole mobility. As mentioned in the introduction the extracted values demonstrate that the CIVIDEC detector is of high quality and a suitable detector for comparison. This analysis cannot be performed on the fabricated planar detector since it is impossible to separately extract electron and hole velocities. This is due to the uncertainty in the location of the generated EHP’s, i.e., they might be at any location in between the electrodes, depending on where the incident alpha passes through the detector region.

The rise time of each type could still be calculated, and provides information about the speed of the detectors. The rise time was found to be $\mathrm {1.22 \pm 0.01 \, ns}$ and $\mathrm {1.34 \pm 0.02 \, ns}$ for the NCN-spiral detectors (CMO13 and CME05). This is only marginally longer than the rise time of the CIVIDEC detector, with a rise time $\mathrm {1.11 \pm 0.01 \, ns}$.

The collected charge was also calculated by integrating under the transient current signal for each operating bias. The integral, INT, is in units of $\textrm{mVns}$ which could be converted into charge using the following equation. This takes into account the input impedance $R=\mathrm {50\,\Omega }$ and gain, $\mathrm {A=150}$, of the amplifier.

1 $$\begin{aligned} Q=\frac{INT}{RA} \end{aligned}$$

**Fig. 2:**
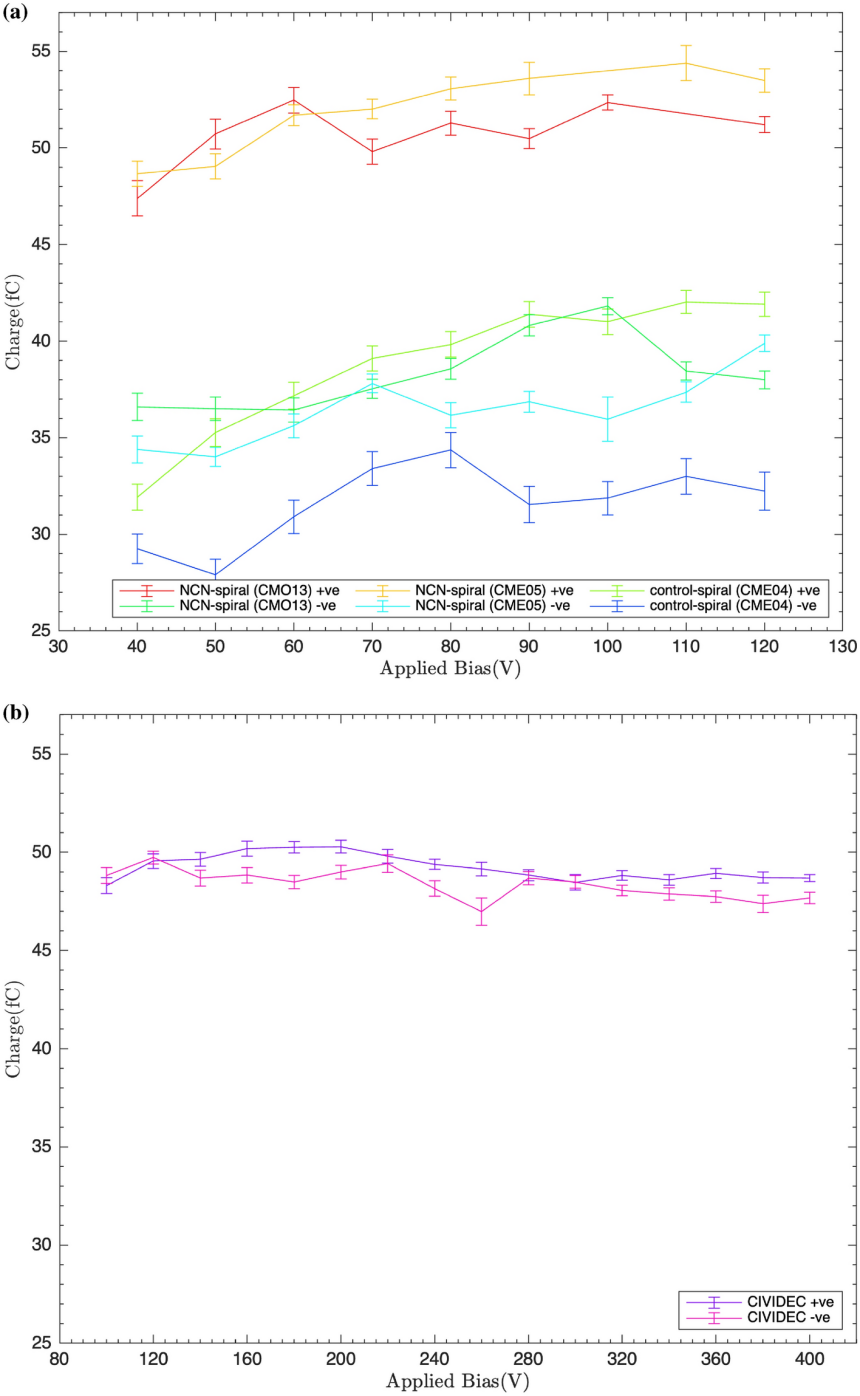
Collected charge as a function of operating bias for (**a**) the spiral detectors and (**b**) the CIVIDEC reference detector.

The charge as a function of applied bias has been produced in Fig. 2a. This was also compared to the CIVIDEC detector charge, depicted in Fig. 2b. The charge collected at 0.8 V μm^−1^, the recommended operating bias of the CIVIDEC detector, is similar (47–49 fC) in both the CIVIDEC and NCN-spiral detectors, corresponding to 400 V and 40 V (using the NCN electrode spacing for the field calculation) respectively.

For the fabricated spiral detectors greater fields, up to 2.4 V μm^−1^, corresponding to 120 V, were applied. This led to greater charge collection. Similarly to the spectroscopy results, the incorporation of NCNs improved the spiral detector charge collection by 25–30%. The maximum charge collected was $\mathrm {42.0 \pm 0.6\,fC}$ at 110V in the control-spiral detector (CME04), compared to $\mathrm {54.4 \pm 0.9 \,fC}$ at 100V for the NCN-spiral detectors (CME05) respectively. Contrary to the spectroscopy results however, a greater charge collection was observed for the NCN-spiral detector (with a positive operating bias) than the CIVIDEC detector, with a maximum charge collection of $\mathrm {54.4 \pm 0.9 \,fC}$ and $\mathrm {50.3 \pm 0.3 \,fC}$ for the NCN-spiral and CIVIDEC detectors respectively.

## Discussion

Four 4 × 4 mm diamond substrates were purchased from Chenguang Machinery and utilised in this work. The nitrogen content of the samples was characterised with Raman spectroscopy. Two samples were used for the control-spiral detectors, this included the substrate with the lowest nitrogen content, and two samples were used for the NCN-spiral detectors.

It was evident that the fabricated spiral detectors required $\mathrm {>13\,h}$ “priming” with the ^241^Am alpha source used in this work, to reach their maximum performance. Therefore, priming was performed on all detectors before attaining the final results.

Overall it is clear that the incorporation of internal NCN “wall” electrodes, within the lateral configured diamond spiral detectors, enhanced the CCE of the detectors by a factor of 9% according to the ^241^Am alpha spectroscopy results and 25–30% according to the ^241^Am alpha induced transient current measurements. This demonstrates that vertical wall-electrodes written into the diamond bulk are effective at projecting electric fields and charge collection.

The detectors were compared with a CIVIDEC MIM detector. When using the CIVIDEC Cx-L Spectroscopic Amplifier for spectroscopy measurements, it was found to have a 6% greater CCE when compared with the NCN-spiral detectors. However the transient current measurements, suggested that the NCN-spiral detectors had comparable CCE to the CIVIDEC detector at 0.8 V μm (40 V for the NCN-spiral detector and 400 V for the CIVIDEC detector). If a larger field, 2 V μm^−1^ (100 V), was applied to the NCN-spiral detector an even greater charge collection was achieved, with a maximum of $\mathrm {54.4 \pm 0.9 \,fC}$. This was larger than the maximum charge collection achieved in the CIVIDEC detector.

It is expected that the shaping output of the CIVIDEC Cx-L spectroscopic amplifier was not well matched to the NCN-spiral detector, leading to loss of some of the signal and an apparent reduction in CCE. This result suggests that full charge collection is achieved with the NCN-diamond spiral detectors despite the substrate containing significant Nitrogen containing defects.

It is also interesting that the NCN-spiral detector behaves differently depending on the polarity of the applied field, with drastically reduced charge collection when a negative field is applied. This has also been observed in other works and is expected to be due to the presence of electrically-active carrier-selective defects at the NCN-diamond interface^32^.

It is expected that further performance enhancements would be realised by reducing the electrode spacing further, and using higher quality material. Future iterations of this device could also include vertical NCN tracks, that allow back contacting of the detector. This would remove the metal contacts from the alpha facing surface, improving the robustness of the device and making it easier to package for *in-situ* monitoring of radiation in corrosive liquid environments, such as in nuclear facilities or for environmental monitoring^33^.

## Conclusion

Three-dimensional planar diamond spiral detectors have been produced which comprise two internal Nano-carbon Network (NCN) wall electrodes, separated by 50 μm, and fabricated using an optimised pulsed fs laser write technology. This approach demonstrates an effective fabrication technique, which results in greatly improved Charge Collection Efficiency (CCE), when compared with surface planar type detectors. Full charge collection was demonstrated, despite using optical grade diamond. Additionally, the detector had a fast response, with $\mathrm {<1.35\, ns}$ rise time.

It is expected that these detectors would maintain their CCE after exposure to large doses of radiation due to their small electrode spacing. As well as offering the prospect of using cheaper, lower quality and more easily sourced, diamond to achieve high performing detectors. Furthermore, it is possible to package these detectors within thicker diamond substrates, which may be required for real-time in-situ monitoring of high pressure aqueous environments, such as the primary cooling loop of a pressurised water reactor. The authors are exploring other geometries such as vertical geometries in thicker diamond windows for such applications.

## Methods

### Substrate characterisation

Four 4 × 4 × 0.3 mm SC diamond substrates (CMO13, CMO14, CME05 and CME04) were procured from Chenguang Machinery. Raman spectroscopy, using a 532 nm laser, was used to determine the quality of the diamond substrates. They all showed evidence of Nitrogen defects, due to the presence of NV peaks and background fluorescence. The NVs have characteristic Raman signatures at $\mathrm {1420\,cm^{-1}}$ (FWHM $\mathrm {=63-65\,cm^{-1}}$) and $\mathrm {3110-3120\,cm^{-1}}$ (FWHM $\mathrm {=47-49\,cm^{-1}}$), which are similar to other works^34^ and were derived from the data in Supplementary S5. It is notable that the fluorescence (characterised by the increasing intensity of the background) is greater when the prominence of the $\mathrm {NV^-}$ is greater , indicating that the substrates contain significant levels of nitrogen, with the exception of CMO14.

**Fig. 3:**
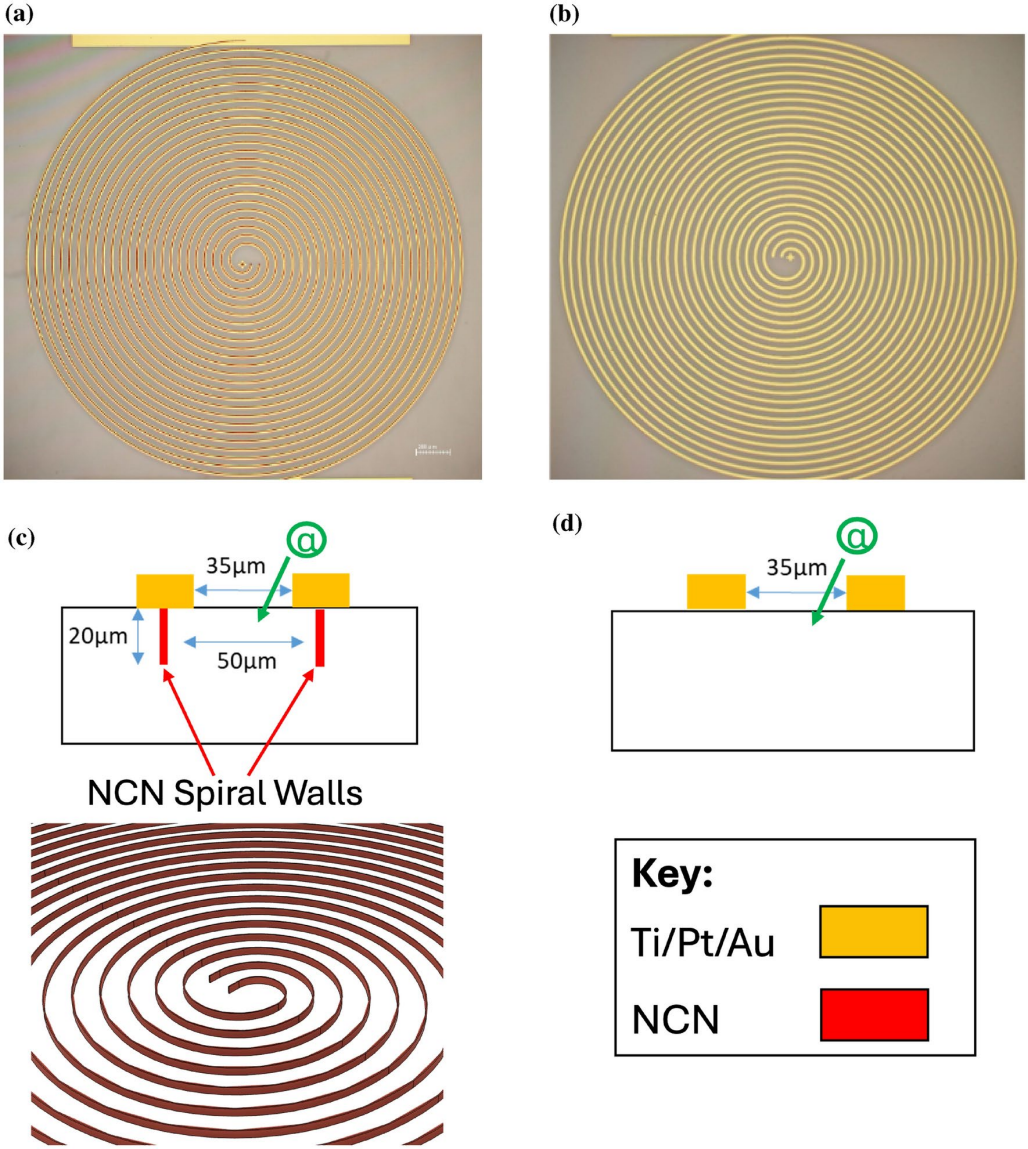
Optical microscope images of the fabricated spiral detectors with and without internal nano-carbon network “wall” electrodes (**a** and **b**), along with a side cross section (**c** and **d**).

Therefore CMO14 and CME04 were used as control samples, and only contained surface Ti/Pt/Au contacts, as depicted in Fig. 3b, d. Referred to as the control-spiral detectors. The other substrates (CMO13 and CME05) also contain internal NCN spiral “wall” electrodes contacted at the surface by Ti/Pt/Au contacts, as depicted in Fig. 3a, c. These are referred to throughout as NCN-spiral detectors.

### Laser writing of nano-carbon network spirals

In the NCN-spiral detectors , the internal spiral NCN “walls” were fabricated using a Ti:Sapphire pulsed fs laser, with $\mathrm {\lambda =790 \,nm}$, 70 nJ pulse energy, 1kHz pulse repetition rate, $\mathrm {0.2 \,mms^{-1}}$ write speed and a tight focusing oil lens (60 x focus and 1.4 numerical aperture). A liquid crystal spatial light modulator (SLM) (Hamamatsu X10468) was used to correct for spherical aberrations arising from refraction at the diamond interface, as described in^26^. First two spiral NCN wire electrodes ($\mathrm {\approx 2 \,\mu m}$ in diameter) were fabricated 20 μm below the diamond surface. To establish a solid base for the three-dimensional spiral, an overwrite was performed before raising the laser focus by 2 μm to fabricate the next spiral layer. This was repeated until the laser focus was approximately 2–3 μm below the diamond surface.

Following this the samples were immersed in an acid etching solution, ${{\text{H}}_2}{\text{S}}{{\text{O}}_4}:{({\text{N}}{{\text{H}}_4})_2}{{\text{S}}_2}{{\text{O}}_8}$, at 200 °C for 20 min, before transferring the sample to an alkaline solution, ${{\text{H}}_2}{{\text{O}}_2}:{\text{N}}{{\text{H}}_4}{\text{OH}}$, for 10 min . This was done to provide an oxygen terminated surface^35^ and clean interface between the NCNs and the Ti/Pt/Au contacts.

### Metal deposition

The surface Ti(50 nm)/Pt(20 nm)/Au(150 nm) contacts were deposited on all 4 substrates and follow the same spiral configuration as the internal NCN “walls”, except they are 15 μm wide resulting in a surface contact separation of 35 μm. A Heidelberg DWL 66+ was used to achieve the spiral pattern by photolithography, the Ti(50nm)/ Pt(20nm)/ Au(150nm) contacts were deposited by electron beam evaporation and lifted off in dimethylsulfoxide.

It should be noted that CMO13 required cleaning and re deposition of the Ti/Pt/Au contact due to damage and the thickness of the contact was approximately 186 nm greater due to thicker deposition of Au.

### Current-voltage characterisation

Prior to exposure to alpha particles the dark current-voltage (IV) characteristics were measured to check the leakage current of the fabricated detectors. A dual sweep was performed from 0 to − 100 V and 0 to 100 V. The current measured is less than 2 *nA* for all the devices.

### Detector characterisation with ^241^Am

Following IV characterisation the detectors were packaged to enable contacting within a custom built alpha detector characterisation facility, termed ALPHIE (Alpha Interaction Experiment). ALPHIE comprises a vacuum chamber($\mathrm {<5 \times 10^{-2} mbar}$), housing a 74kBq $\mathrm {^{241}Am}$ sealed alpha source, separated from the detector stage by 35mm. The detector is connected to a CIVIDEC ROSY to the external electronics via an internal coaxial cable and vacuum feed-through. Alpha spectroscopy and transient current measurements were performed on the CIVIDEC MIM and fabricated spiral detectors.

Due to the presence of N defects, it was found that the fabricated spiral detectors required alpha priming to reach their maximum CCE. Alpha priming involves filling deep traps by irradiating the sample with high radiation doses^5^ and was first reported in 1998^36^. Spectra demonstrating this effect for both the NCN and control-spiral detectors can be found in S1 and S2.

First alpha spectroscopy measurements were performed using the CIVIDEC Cx-L spectroscopic amplifier, which is a charge sensitive amplifier, with shaping output.

The CIVIDEC MIM detector is operated with a 400V bias, corresponding to a field of $\mathrm {E = 0.8 \, V \mu m^{-1}}$ and alpha spectra were acquired for 5 mins every 22 mins for a period of $> 22$ hours, switching the bias off after each acquisition. In this time little change was observed in the spectra.

The fabricated spiral detectors were operated at 100V. A 5 min spectra was acquired every hour (for CME05) or 22 mins (for all others) for $> 25$ hours. A Matlab peak fitting tool (findpeaks) was used to determine the point at which the CCE was at a maximum and the summed spectra after this point were used for comparison.

The CIVIDEC Cx-L spectroscopic amplifier contains a fixed shaping time and does not allow analysis of the rise time of the incoming charge pulses. Thus the C2-TCT Broadband Amplifier, a current sensitive amplifier with no shaping output, was used to measure the raw unshaped signal, and the rise time and total charge collection were extracted.

## Supplementary Information

Supplementary Information.

## Data Availability

The datasets generated during and/or analysed during the current study are available from the corresponding author on reasonable request.
