# Supplementary material for: Three-dimensional diamond planar spiral detectors
Source: Sci Rep. 2025 Mar 12;15:8496. doi: 10.1038/s41598-025-93332-7 (PMC11904232; doi:10.1038/s41598-025-93332-7)
Supplement: Supplementary file 1 — Supplementary Information. [file 41598_2025_93332_MOESM1_ESM.pdf]

Supplementary Information

(a)

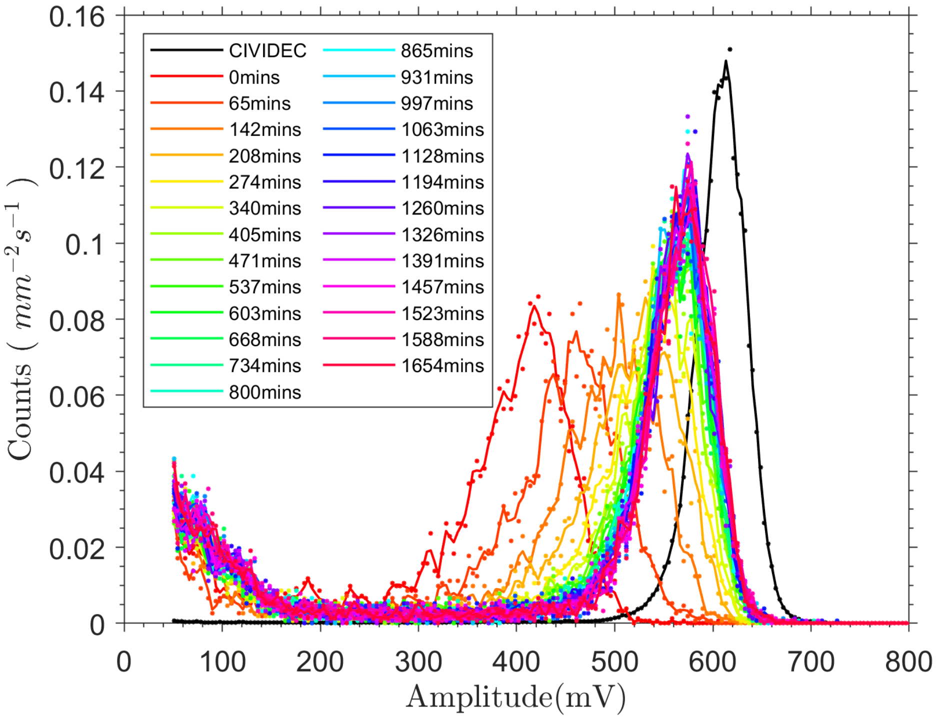

(b)

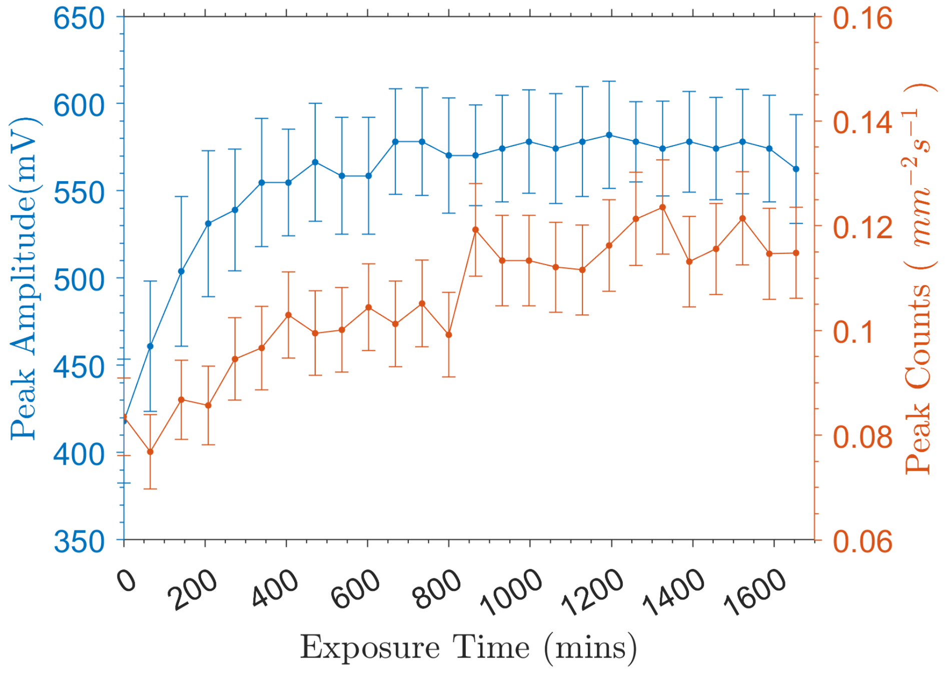

Figure S1: In a) the ^{241}Am alpha spectra from a NCN-spiral detector (CME05), operated at 100V, with varying alpha exposure time are presented. In b) the alpha primary peak location (amplitude) and intensity (counts) have been determined and plotted against alpha exposure time. It should be noted that '0mins' cannot be taken as the unprimed spectrum, each spectral acquisition was 5 mins and the bias was switched off between acquisitions.

(a)

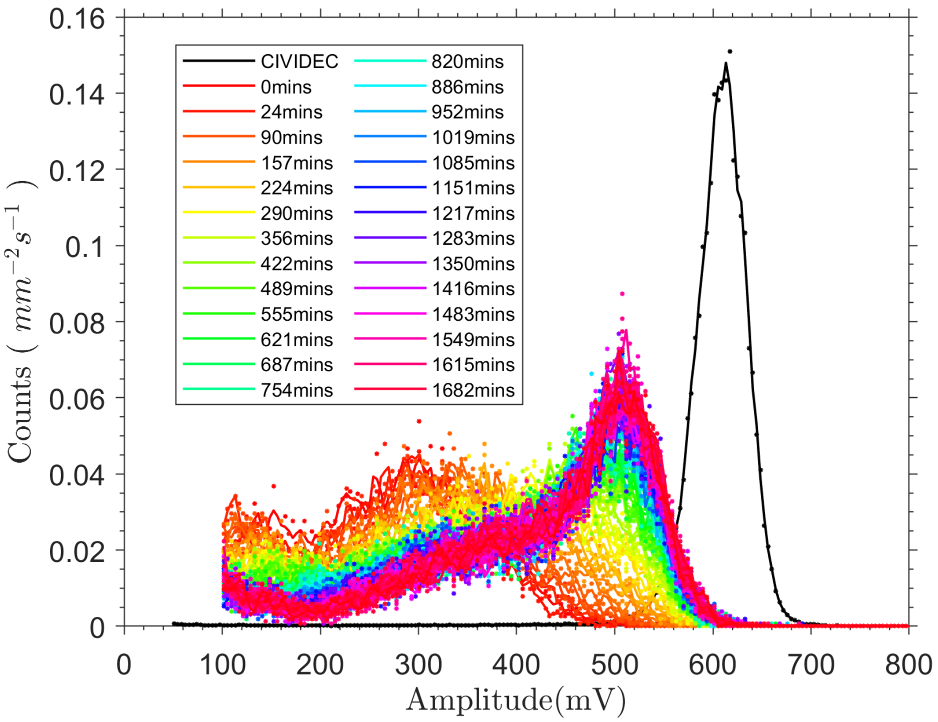

(b)

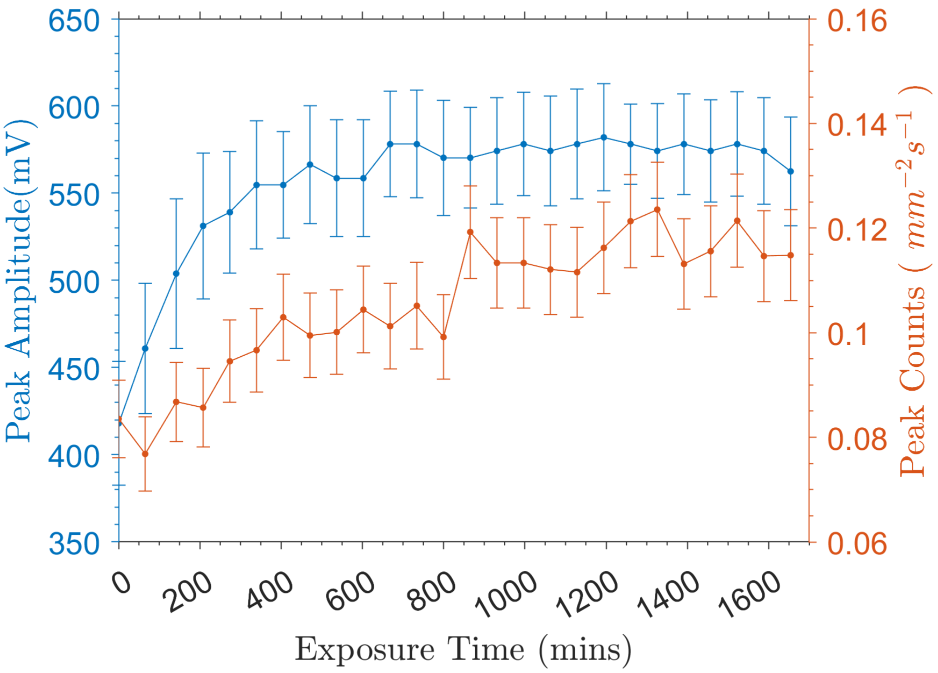

Figure S2: In a) the ^{241}Am alpha spectra from a spiral detector without NCNs (CME04), operated at 100V, with varying alpha exposure time are presented. In b) the alpha primary peak location (amplitude) and intensity (counts) have been determined and plotted against alpha exposure time. It should be noted that '0mins' cannot be taken as the unprimed spectrum, each spectral acquisition was 5 mins and the bias was switched off between acquisitions.

(a)

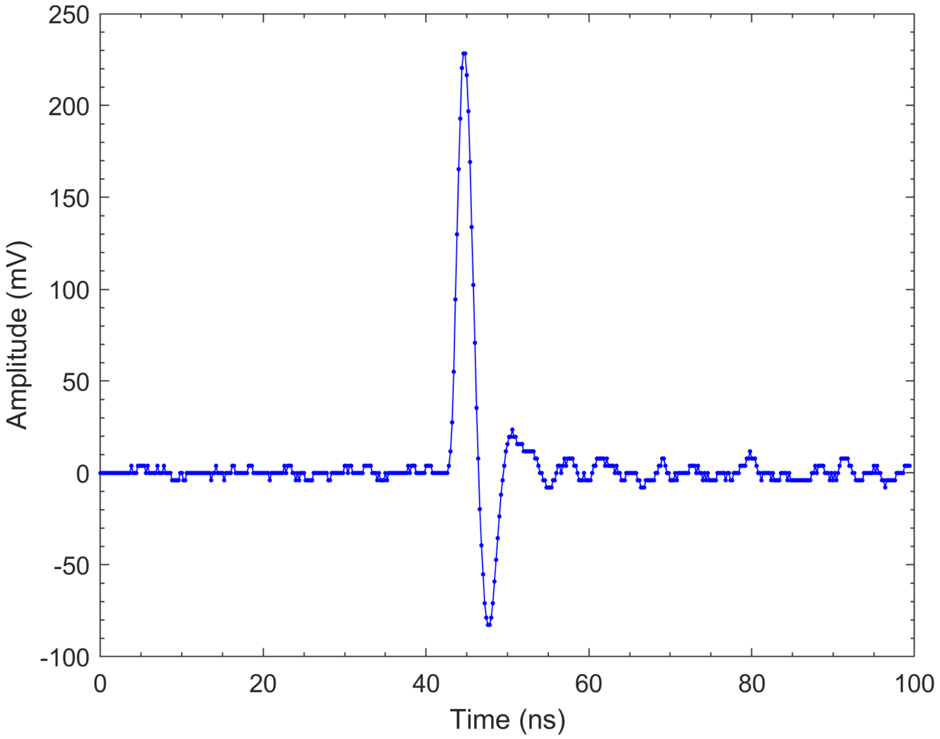

(b)

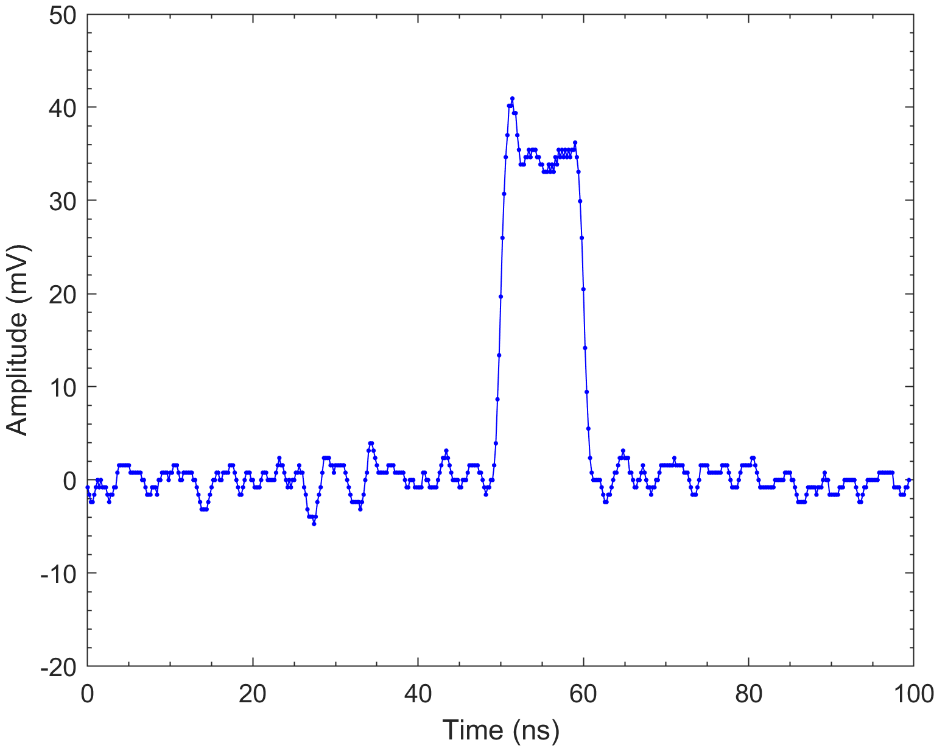

Figure S3: A representative transient current pulse for a) a NCN-spiral detector (CMO13) and b) the CIVIDEC reference detector.

(a)

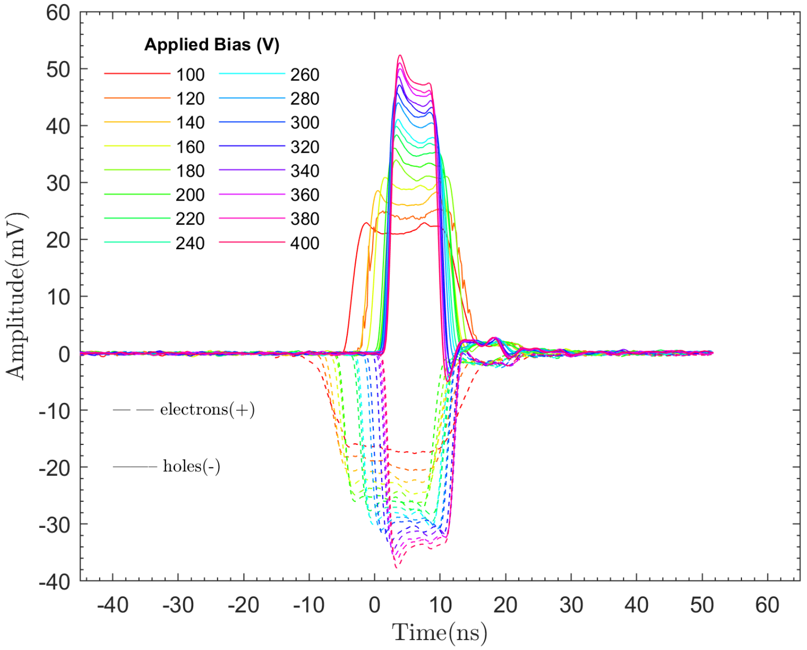

(b)

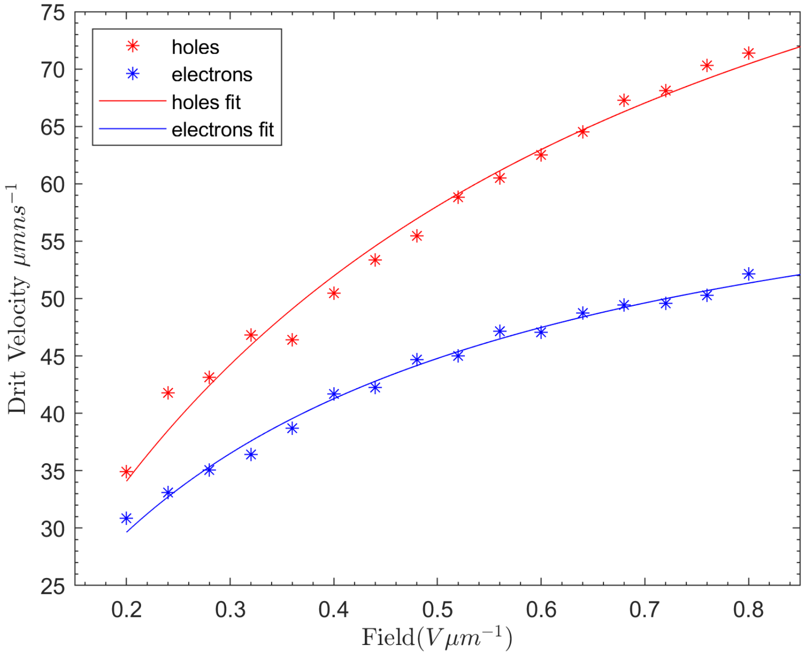

Figure S4: a) ^{241}Am alpha induced transient current signals measured at various operating bias for the CIVIDEC B3 Spectroscopic detector. The drift velocity, $\mathrm{v_d}$ can be derived from a). $\mathrm{v_d} \approx \frac{\mathrm{d}}{\mathrm{t_c}}$, where d is the detector thickness and $\mathrm{t_c}$ is the charge cloud transit time, which can be derived from the FWHM of the transient current plot [?]. This was then plotted against the applied field in b). The equation, $\mathrm{v_d} = \frac{\mu_0 \mathrm{E}}{1+\mu_0 \mathrm{E}/\mathrm{v_{SAT}}}$, proposed in [?], has been fitted to the data, to allow extraction of the electron mobility, hole mobility, electron saturation velocity and hole saturation velocity. These were found to be $\mu_{0\mathrm{h}} = 2500 \pm 200\,\mathrm{cm^2V^{-1}s^{-1}}$, $\mu_{0e} = 2600 \pm 200\,\mathrm{cm^2V^{-1}s^{-1}}$, $\mathrm{v_{sath}} = (1.09 \pm 0.08) \times 10^7\,\mathrm{cms^{-1}}$ and $\mathrm{v_{sate}} = (6.8 \pm 0.2) \times 10^6\,\mathrm{cms^{-1}}$ respectively.

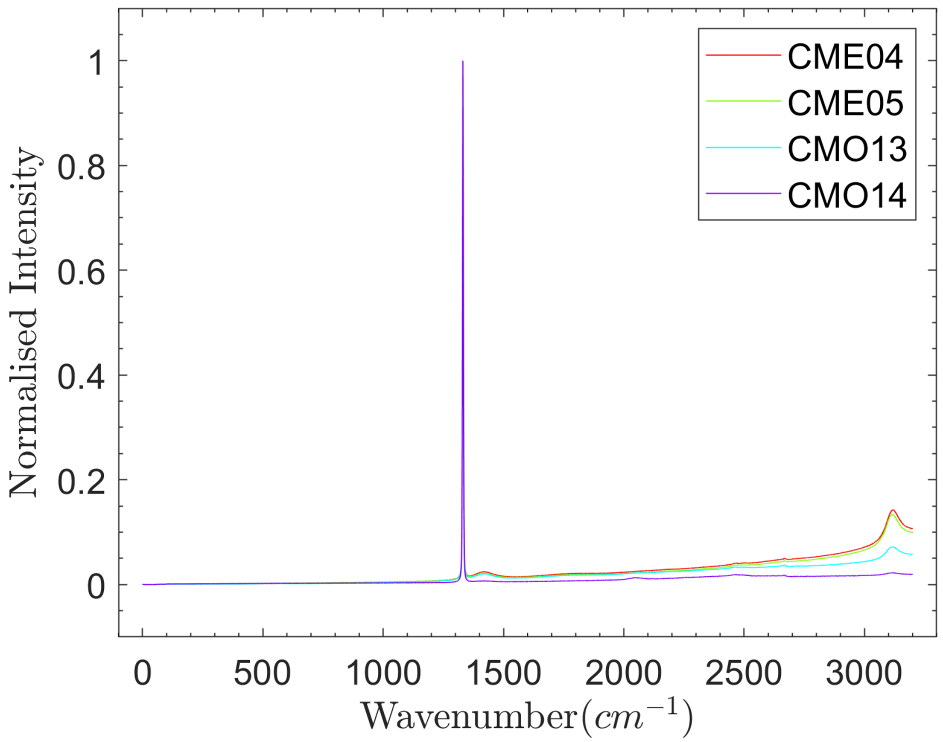

Figure S5: Raman spectra, normalised to the 1332cm^{-1} diamond peak, showing the difference in quality between the four diamond substrates used to fabricate the spiral detectors in this work. NV^0 and NV$^-$ can be found at $1420cm^{-1}$ (FWHM $= 63 - 65cm^{-1}$) and $3110 - 3120cm^{-1}$ (FWHM $= 47 - 49cm^{-1}$).
